# Neoadjuvant chemotherapy for breast cancer: an evaluation of its efficacy and research progress

**DOI:** 10.3389/fonc.2023.1169010

**Published:** 2023-10-03

**Authors:** Yushi Chen, Yu Qi, Kuansong Wang

**Affiliations:** ^1^ Department of Pathology, Xiangya Hospital, Central South University, Changsha, Hunan, China; ^2^ Department of Pathology, Basic Medical School, Central South University, Changsha, Hunan, China

**Keywords:** breast cancer, neoadjuvant chemotherapy, treatment, evaluation, imaging technique

## Abstract

Neoadjuvant chemotherapy (NAC) for breast cancer is widely used in the clinical setting to improve the chance of surgery, breast conservation and quality of life for patients with advanced breast cancer. A more accurate efficacy evaluation system is important for the decision of surgery timing and chemotherapy regimen implementation. However, current methods, encompassing imaging techniques such as ultrasound and MRI, along with non-imaging approaches like pathological evaluations, often fall short in accurately depicting the therapeutic effects of NAC. Imaging techniques are subjective and only reflect macroscopic morphological changes, while pathological evaluation is the gold standard for efficacy assessment but has the disadvantage of delayed results. In an effort to identify assessment methods that align more closely with real-world clinical demands, this paper provides an in-depth exploration of the principles and clinical applications of various assessment approaches in the neoadjuvant chemotherapy process.

## 1 Introduction

Breast cancer (BC) incidence continues rising, being the leading cause of cancer death in women in the last Global Cancer Statistics 2020 (1). Apart from the traditional surgical plus adjuvant therapies, neoadjuvant chemotherapy (NAC) has been increasingly applied. Breast cancer neoadjuvant chemotherapy refers to systemic chemotherapy before planned surgical treatment or local treatment of surgery plus radiotherapy for newly treated breast cancer patients who have not found distant metastasis. It aims to transform initially inoperable tumors into operable ones, providing patients with the opportunity for surgery, enhancing breast-conservation rates. Concurrently, it allows for the assessment of the tumor’s sensitivity to drugs, guiding the patient’s subsequent treatment options (2). Neoadjuvant chemotherapy, as an essential part of breast cancer treatment, is still in a stage of continuous development (3). In the past, NAC was reserved for patients with locally advanced or inoperable breast cancer with the primary purpose to reduce the tumor size (also known as downstaging) to allow breast-conservation surgery and possibly omit axillary dissection in patients who are opposed to an extensive operation. However, currently the role of NAC has expanded to include patients with early-stage, operable breast cancer. As various clinical trials and new treatment concepts continue to emerge, its treatment mode has also changed from the single chemotherapy to the current neoadjuvant chemotherapy which based on different molecular subtypes of breast cancer, such as neoadjuvant anti-human epidermal growth factor receptor 2 (HER-2) targeted therapy combined with chemotherapy and neoadjuvant endocrine therapy (4). Overall, NAC improves the outcome of breast cancer treated with surgery (5, 6). It is supposed that the combination of NAC with traditional treatment will bring the best benefits to patients in the research field (7).

Nowadays, undergoing surgery after a successive combination of drugs is considered the gold standard for assessing tumor response (8, 9). However, not all BC patients benefit from the NAC setting and, therefore, it is critical to differentiate between the subjects that will respond positively and those who will not, in order to choose alternative and more effective therapies. With the continuous enrichment of new evidence-based medicine data, the differences between various treatment concepts and clinical practices have become more apparent. How to choose neoadjuvant treatment indications in clinical practice, optimize patients’ treatment strategies, and improve treatment outcomes is still controversial. Neoadjuvant chemotherapy exerts a crucial effect on the comprehensive treatment of breast cancer, but the prediction of efficacy is not perfect (10). Factors such as tumor size, histological type, differentiation status, tumor-associated lymphocytes, and immunohistochemical marker status affect the clinical response to neoadjuvant chemotherapy. Moreover, chemotherapy response is an independent predictor of overall treatment outcome, so a reliable method is needed to predict early results of neoadjuvant chemotherapy treatment.

**Figure 1:**
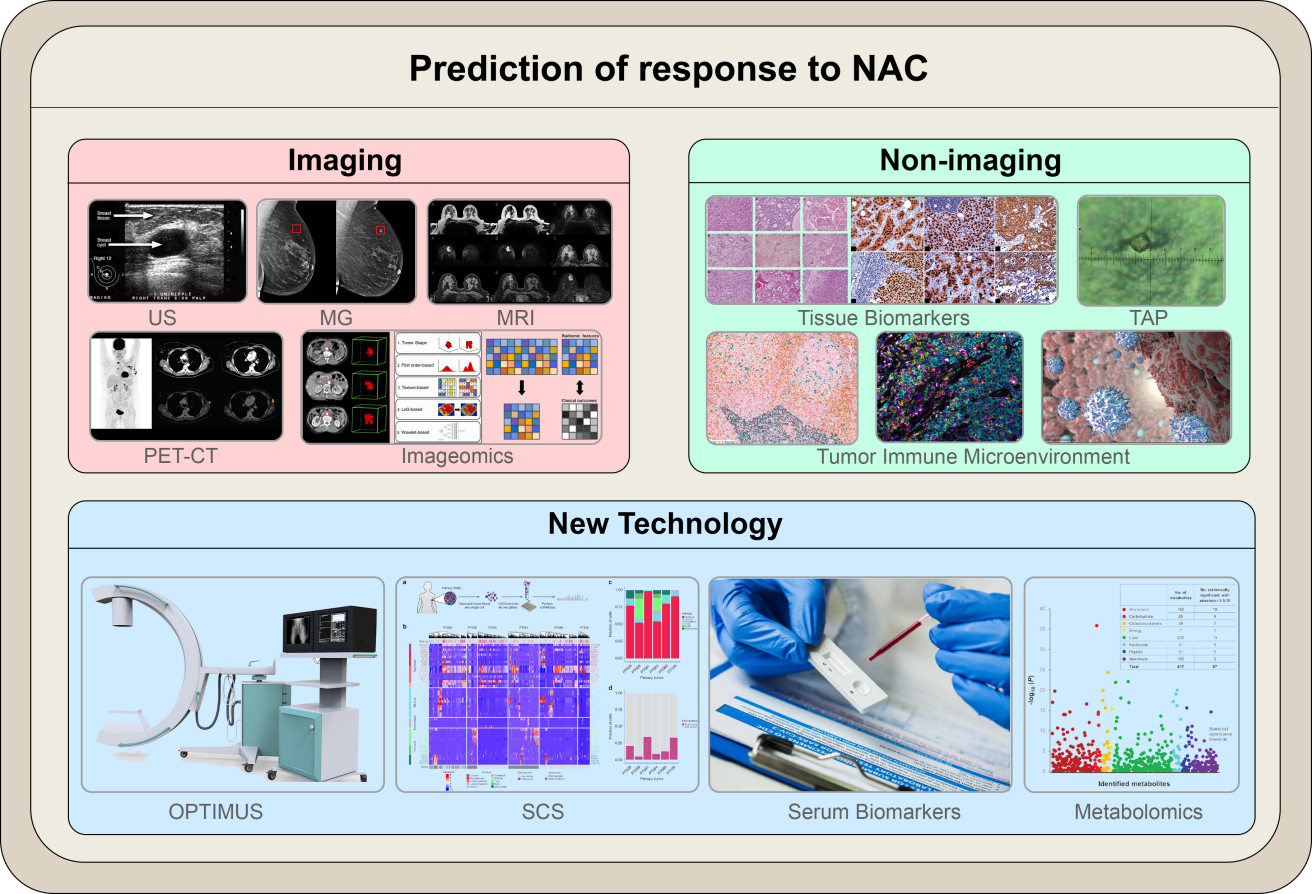
The illustration summarizes the modalities outlined in the article to predict response to NAC. Novel technologies that utilize the molecular characteristics of cancer cells are currently being investigated; these include both molecular imaging and non-imaging profiles. Molecular-based techniques are expected to replace conventional anatomical-based techniques in predicting responses to NAC in breast cancer.

The current clinical evaluation methods for the efficacy of neoadjuvant chemotherapy for breast cancer are mainly divided into two categories: clinical imaging evaluation and non-imaging (micro-pathological) evaluation (**Figure 1**).

Clinical examination combined with conventional imaging modalities, containing magnetic resonance imaging, computed tomography, ultrasound, and mammography, has been utilized to predict and assess tumor response to neoadjuvant chemotherapy (11). Nevertheless, when assessing tumor anatomical and morphological features using these imaging techniques, a delay between assessment of initial tumor size and shrinkage may be detrimental to distinguishing viable tumors from fibrotic scar tissue. Before NAC, ultrasound (US or sonography or echography) of the breast should be performed to document tumor size. Sometimes, magnetic resonance imaging (MRI) may be helpful in calculating tumor volume and extent in the breast and making decisions on whether to undergo breast-conserving surgery, in addition to the diagnostic capability of breast tumor at the time of the first diagnosis (12). Once a patient has completed NAC, imaging tests should be performed to guide the surgical approach. US imaging of the involved breast and ipsilateral axilla is usually required. Additionally, MRI may be obtained if the tumor is not well visualized on US or if a better definition of the extent of tumor would assist the surgeon in determining the optimal surgical approach depending on the institution. In patients considering breast-conserving therapy, pretreatment or repeated US and MRI have been clinically important to appropriately assess the presence of tumor distribution. Furthermore, several studies indicated that the presence of tumor after NAC drawing support from histopathological examination of the tumor bed could be the golden standard as well as pCR rate. Further surrogate biomarkers could be applied for evaluating the outcomes defined by Miller-Payne system following neoadjuvant settings (8). Fine-needle aspiration cytology is considered as an accurate technique performed by experienced cytologists to assess the existence of breast cancer (13). The core breast biopsy used to be a traditional examination technique to identify the initial diagnosis of breast lumps. Fortunately, this critical technique is able to accurately predict pathologic responses after NAC (14). In previous studies, biomarker changes before and after NAC were claimed to have great clinical relevance to age or grade impacts (15). For example, ER and Ki-67 status were reported to possess obvious changes after NAC treatments in breast cancer patients. Regarding NAC ‘s efficacy, recent studies tackle the relationship between BC phenotypes and treatment outcomes (16–18), revealing pathological complete response (pCR) as a surrogate biomarker of response and survival (19, 20). Nevertheless, this procedure is invasive and time consuming. Thus, faster, less invasive and more sensitive tools are required in order to detect useful molecular and/or clinical predictors of pCR (21, 22). This paper will review the clinical application of various assessment methods in neoadjuvant chemotherapy and new technological advances.

## 2 Macroscopic imaging

The imaging detection of breast cancer can objectively provide specific values and then evaluate the condition of tumor lesions from different aspects. Due to the non-invasive characteristics of the operation process, imaging evaluation has become the most common method for clinical evaluation of the treatment effect during breast cancer NAC (23). Commonly used imaging evaluations of breast cancer NAC include: color Doppler ultrasound, mammography, nuclear magnetic resonance, positron emission tomography.

### 2.1 Ultrasound technology

With the continuous advancement of diagnosis and treatment methods, the position of ultrasound (US) technology in evaluating the efficacy of breast cancer NAC has become more and more critical. Multi-modal ultrasound technology can provide in-formation on breast tumor size, morphology, blood supply, and other aspects and can further show the internal and surrounding blood flow perfusion, determine whether the lump is liquefaction and necrosis, and so on (24). Due to its advantages in terms of ease of management, convenient operation, and lack of radiation risks, Multi-modal ultrasound imaging technology has broad application prospects in breast cancer NAC. It can conduct the multi-parameter and overall evaluation of the efficacy of neoadjuvant chemotherapy for patients to guide the clinic better and improve patients’ prognoses.

Currently commonly used clinical ultrasound techniques include: conventional ultrasound, color Doppler ultrasound, contrast-enhanced ultrasound, elastography, and automatic breast full-volume imaging. We have introduced and analyzed each ultrasound imaging method, outlining their respective advantages and limitations.

#### 2.1.1 Conventional ultrasound

Conventional grey-scale ultrasound provides information on the morphology and internal structure of the tumor post-NAC, and its relationship with surrounding tissues, particularly noting tumor size changes (25). However, the specificity and accuracy of conventional ultrasound for NAC efficacy assessment are relatively low due to factors such as operator error and selection of cut surfaces (26). For instance, a decrease in cancer cell count under the microscope post-NAC does not always reflect as tumor shrinkage macroscopically. Instead, some lesions may exhibit honeycomb shrinkage, leading to challenges in defining lesion boundaries and accurately measuring tumor size. These limitations mean that conventional ultrasound must be supplemented with other imaging and pathology methods to ensure accurate assessment.

#### 2.1.2 Color Doppler flow imaging

Breast cancer is a vascular-dependent disease. Its growth, infiltration, and metastasis are intrinsically linked to the formation of new blood vessels, particularly micro-vessels (27, 28). Research has established that changes in these tumor micro-vessels during neoadjuvant chemotherapy (NAC) for breast cancer often precede morphological alterations. NAC has the capacity to influence the blood supply to tumor cells, leading to cell death and modifications to the blood flow speed and resistance within and surrounding the lesion. Color Doppler Flow Imaging (CDFI) has proven to be an effective tool in mirroring the therapeutic impact of NAC in breast cancer, achieved by detecting hemodynamic shifts in the lesion area (29). Consequently, it has significant implications for the clinical evaluation of NAC’s therapeutic effect. With the aid of high-frequency probes, CDFI can produce high-resolution images and exhibit exceptional sensitivity, allowing for clear delineation of the tumor boundary and detailed representation of its microstructure and size. However, it’s worth noting that CDFI also has its constraints. For example, it can only provide a limited cross-sectional view of the tumor, hindering a comprehensive assessment of tumor shrinkage. Additionally, it lacks the ability to differentiate between residual tumor and fibrotic tissue resulting from the chemotherapy reaction (30). While CDFI is capable of an accurate assessment of breast cancer NAC efficacy in the early stages, it’s restricted to displaying blood flow signals with a diameter larger than 0.2 mm and relatively high flow rates. This constraint limits its sensitivity to display the tumor’s micro-vessels (31). Consequently, providing a complete picture of tumor vasculature proves challenging, which could lead to an insufficient evaluation of breast cancer’s blood supply. Therefore, despite its strengths, the overall capability of CDFI in evaluating NAC’s curative effect remains limited.

#### 2.1.3 Contrast-enhanced ultrasonography

The principle of Contrast-enhanced ultrasonography (CEUS) is to use the nonlinear effect of the gas microbubbles in the blood in the sound field and the strong backscatter produced to obtain contrast-enhanced images (32). Several studies have shown that, compared with conventional ultrasound, CEUS can objectively show the blood flow perfusion in tumors, has the advantages of safety and convenience, and can more accurately evaluate the efficacy of breast cancer NAC (33–35). However, CEUS has certain limitations for tumors with less vascular distribution or tumors with a deep location, and it cannot well show the characteristics of its microvascular structures and microcirculation.

#### 2.1.4 Ultrasound elastography

Ultrasound elastography (UE) is a new method for measuring tissue hardness proposed by Ophir J, and it is an essential supplement to conventional ultrasound (36). Studies have confirmed that the higher the hardness value of breast tumor-infiltrating foci, the worse the prognosis. After receiving NAC, the state of the tumor’s internal and surrounding tissues can be changed by the action of drugs, and its hardness will also change accordingly (37). UE can use real-time color images to reflect the difference in tissue deformation between the diseased area and the surrounding normal tissues when external forces compress them and use related technologies to quantitatively evaluate the hardness of the tissues, which has a high degree of specificity and sensitivity in the differential diagnosis of benign and malignant lesions (38). Compared with conventional ultrasound, UE has higher sensitivity in evaluating the efficacy of NAC. However, it also has limitations, such as UE’s higher operator requirements, high image quality susceptible to patient status (such as breathing, fat layer thickness, peripheral vascular beats), and not being fully sampled for larger lumps. The continuous development of UE technology has enormous potential in evaluating the efficacy of breast cancer NAC.

#### 2.1.5 Automatic breast volume scanner

The Automatic Breast Volume Scanner (ABVS) represents a technological advancement in the field of breast disease examination. This three-dimensional ultrasound imaging tool enables automatic, full-volume, and coronal scanning of the breast (39). The three-dimensional reconstruction offered by ABVS allows a complete dynamic display of the coronal surface of breast tumors. This feature provides a more accurate representation of tumor growth and a clearer understanding of the mass’s relationship with surrounding tissues (40).

ABVS standardizes image data storage and enhances the precision of stereoscopic multi-section images through post-image processing technology (41). It rectifies the traditional limitations of ultrasound such as repeatability and operator dependence, compensating for the absence of volume imaging in CDFI and enhancing its value in diagnosing and treating breast disease.

The Breast Imaging Reporting and Data System (BI-RADS), developed by the American College of Radiology (ACR) in 2003, serves as a grading evaluation system to assess breast ultrasound images. The system categorizes the mammogram results from 0 through 6, as detailed in **Table 1**. It outlines the features of breast ultrasound images considering blood vessel distribution, lesion calcification, masses, and echo structures (42, 43). This system is instrumental in standardizing diagnosis results, monitoring lesion tissue over time, comprehensively evaluating clinical efficacy, and selecting quantitative computer features. Studies suggest that combining ABVS with BI-RADS can yield a more comprehensive and standardized evaluation of NAC treatment in breast cancer (44). Despite being in its exploratory stage, ongoing developments in ultrasound imaging technology suggest a promising role for ABVS in the clinical diagnosis and treatment of breast cancer NAC.

**Table 1:** The interpretation of the Bread Imaging Reporting and Data System (BI-RADS).

Categories	Mammograms’ results	Treatments
Category 0	No findings	Additional imaging evaluation and/or comparison to prior mammograms is needed.
Category 1	Negative	No treatments.
Category 2	Benign(noncancerous) finding	No treatments.
Category 3	Probably benign finding	Follow-up in a short time frame is suggested.
Category 4	Suspicious abnormality	Biopsy should be considered.
Category 5	Highly suggestive of malignancy	Appropriate action should be taken.
Category 6	Known biopsy-proven malignancy	Appropriate action should be taken.

#### 2.1.6 Superb microvascular imaging

Superb microvascular imaging (SMI) is a new microvascular ultrasound imaging technology. With the color Doppler principle, the difference between micro-vessels and normal low-speed moving tissues is extracted, and the micro-vessels with low blood flow velocity can be detected with high frame rate and high resolution, and low-velocity micro-vessels with a diameter of >0.1 mm can be displayed (45). SMI can detect low-velocity blood flow and micro-vessels and reduce the influence of tissue motion clutter. It can image the micro-vessels of the lesion without the need for contrast agents and display micro-vessels non-invasively and sensitively (46). Therefore, SMI can early display the microvascular status of breast cancer, assess whether chemotherapy is effective, help clinical change of the treatment plan in time, improve the chemotherapy effect, and judge the prognosis. It is an effective inspection method for evaluating the efficacy of breast cancer NAC.

### 2.2 Mammography

Mammography (MG) is a commonly used X-ray examination method for diagnosing breast cancer. This method is less expensive, painless, and has a high sensitivity for breast cancer diagnosis, especially carcinoma *in situ* (47). Mammography is more sensitive to microcalcification of breast lesions, and the detection rate of breast cancer is relatively improved (48). It can be compared with the mammography performance of breast masses before and after NAC, from the shape, size, calcification range, density, and axillary lymph nodes. The size and other changes are used to evaluate the efficacy of NAC (49). Studies have shown that MG has low accuracy in assessing lesions, has a poor display effect on lesions in dense breasts (50), and has radiation damage, but due to its wide application, it is still one of the primary screening methods used by most hospitals to evaluate the efficacy of breast cancer NAC.

### 2.3 Magnetic resonance imaging

Breast magnetic resonance imaging (MRI) is the most sensitive and accurate examination method for evaluating the curative effect of breast cancer NAC. It has many evaluation indicators and is not affected by the density of glands. Because MRI demonstrates a higher detection rate for multi-center, multi-lesion conditions compared to traditional imaging methods (51). Compared with mammography, ultrasound, and other inspection methods, MRI can use different imaging techniques to evaluate the response to NAC treatment early and comprehensively from the aspects of morphology, hemodynamics, and metabolism.

#### 2.3.1 Conventional magnetic resonance imaging

Tumor diameter and tumor volume are currently the most commonly used indicators for evaluating the efficacy of breast cancer NAC. Studies have shown that reducing tumor volume is the most accurate indicator of pathological response after treatment, followed by the change of tumor diameter (52). Compared with other imaging methods, MRI has higher sensitivity and accuracy in evaluating tumor size, and it has a good correlation with the final pathological evaluation results (51). In addition to the preliminary measurement of the size of the lesion, conventional MRI has a higher judgment value for tumor fibrosis after NAC and can make a preliminary imaging diagnosis of the relationship between the tumor and the surrounding tissues.

Nevertheless, conventional MRI may overestimate the extent of residual disease following NAC, especially in scenarios characterized by substantial inflammation or fibrosis. This may precipitate unnecessary surgical interventions. Additionally, its sensitivity towards smaller residual diseases is somewhat limited (51). Thus, to complement the limitations of conventional MRI, the application of functional MRI techniques, such as diffusion-weighted imaging and dynamic contrast-enhanced MRI, becomes imperative. These advanced imaging methodologies can provide additional insights into the biological and physiological characteristics of the tumor, enhancing the precision and accuracy of the assessment.

#### 2.3.2 Dynamic contrast-enhanced magnetic resonance imaging

Dynamic contrast-enhanced magnetic resonance imaging (DCE-MRI) is the most commonly used hemodynamic examination method, which can show the structural characteristics of the tumor and surrounding tissues in detail, detect tumor angiogenesis, and is the most sensitive detection method for breast cancer (53). The principle of this technique is to use continuous and rapid imaging methods to obtain semi-quantitative or quantitative parameters by acquiring images before and after the contrast agent injection and through calculation and analysis (54). The microvascular system in the diseased tissue is used as the physiological basis to evaluate the physiological properties of the diseased tissue. Compared with conventional MRI, this method can obtain the morphological feature information of the lesion and reflect the physiological changes of the lesion (55). Studies have confirmed that DCE-MRI analyses the density, integrity, and permeability of blood vessels in tissues through various parameters clarifies the biological changes within the tumor and thus plays a role in evaluating the efficacy of breast cancer NAC.

Of note, DCE-MRI requires the administration of a contrast agent, which can engender complications in a small subset of patients, including allergic reactions and nephrogenic systemic fibrosis. This hampers its applicability in patients with compromised renal function.

#### 2.3.3 Diffusion-weighted imaging

In malignant tumors, the cell density increases, the normal structure of the cells are destroyed, and the movement of water molecules in the cell microenvironment is hindered. Chemotherapy drugs can kill tumor cells, reduce cell density in tumor tissue, increase tissue gaps, and have faster-moving molecules (56). Therefore, the use of MR imaging parameters to evaluate the changes in water molecule movement before and after NAC can also reflect the treatment effect of the tumor early and predict the long-term prognosis. Diffusion-weighted imaging (DWI) is an MR imaging method based on the Brownian motion of water molecules in the tissue. Its leading indicator is the apparent diffusion coefficient (ADC), which can quantify the diffusion motion of water molecules, proportional to the diffusion rate of water molecules in tissues, and visualize the strength of water molecule motion in tissues (51). Studies have shown that DWI can early detect NAC treatment response through the degree of diffusion of water molecules, tumor cell structure, and cell membrane integrity (57). However, the DWI sequence has certain limitations, its spatial resolution is low, there are differences in ROI delineation, and the sensitivity of breast cancer with different molecular subtypes is different. The accuracy of using pre-chemotherapy ADC values alone to predict the NAC response of breast cancer needs to be further evaluated clinically.

#### 2.3.4 Intravoxel incoherent motion diffusion-weighted imaging

Intravoxel incoherent motion diffusion-weighted imaging (IVIM-DWI) is a new technology developed based on DWI, which can simultaneously reflect the diffusion of water molecules in the tissue and the perfusion of microcirculation, which has more advantages than traditional DWI. Its parameters are Perfusion fraction (f), which reflects the proportion of microcirculation perfusion-related dispersion in the total dispersion in the voxel, and its size is related to blood volume. Diffusion coefficient (D) refers to the diffusion of pure water molecules. Perfusion-related diffusion coefficient (Pseudo diffusion coefficient, D∗) is diffusion coefficient related to capillary perfusion (58). Studies have shown that the IVIM model can reflect the diffusion of water molecules in the tissue and the microcirculation perfusion, thereby distinguishing the benign and malignant breast tumors, and has potential value in the molecular classification of breast cancer, prognostic factors, and the evaluation of chemotherapy effects (56). However, IVIM is still controversial in the academic circles regarding the specification of breast cancer scanning parameters, improved image analysis models, and whether its D value and f value can predict the therapeutic effect of malignant tumors before NAC (59). Therefore, whether the IVIM model can be routinely applied in clinical practice requires further research and verification.

### 2.4 Positron emission tomography - computed tomography

PET-CT imaging in breast cancer provides essential information regarding morphological changes in breast lesions and lymph nodes pre- and post-neoadjuvant chemotherapy (NAC). It has been substantiated through research that PET-CT plays a critical role in assessing NAC’s effectiveness in treating breast cancer. It exhibits higher sensitivity, specificity, and accuracy in assessing residual lesions, only surpassed by MRI (60). Furthermore, it offers high specificity in evaluating axillary lymph nodes, favoring the clinical application of sentinel lymph node biopsy over axillary dissection (61). Nevertheless, PET-CT faces certain limitations, such as reduced spatial resolution, which may lead to an underestimation of invasive lobular carcinoma and *in situ* intraductal carcinoma (62). Moreover, due to its high radiation dose, it is not viable for routine assessment of NAC’s effectiveness in breast cancer patients.

### 2.5 Imageomics

The high heterogeneity of tumors poses challenges in assessing tumor response after chemotherapy using a single imaging index or parameter. Recently, the convergence of big data and medical imaging-assisted diagnostic technology has led to the emergence of a novel image analysis method known as imageomics. This technique extracts extensive features from images to quantify the characteristics of critical diseases like tumors, thereby effectively addressing the complexities arising from tumor heterogeneity (63). Contrary to traditional medical image analysis, imageomics does not necessarily rely on visual interpretation of images. Instead, it employs advanced statistical analyses and computations, combining these data with other clinical data from patients to analyze, extract, and sort through vast amounts of data. The goal is to identify relevant factors that could enhance diagnostic accuracy, prognostic evaluation, and efficacy prediction (64). Given these strengths, the use of imageomics for the analysis of high-level tumor features can effectively address the limitations of traditional morphological assessments of tumor heterogeneity. Furthermore, imageomics can evaluate the efficacy of NAC and even predict tumor prognosis prior to the occurrence of morphological changes.

## 3 Microscopic pathology

Despite advancements in clinical imaging evaluation techniques, the gold standard for assessing the efficacy of neoadjuvant chemotherapy for breast cancer remains the microscopic examination of pathology. The pathologic evaluation of neoadjuvant chemotherapy for breast cancer is based on microscopic changes in the postoperative lesion, which is delayed compared to clinical evaluation and can only be performed after resection of the specimen and cannot be monitored dynamically (23). Pathologic complete response (pCR) can be used as an alternative prognostic endpoint in neoadjuvant drugs for breast cancer clinical trials. Achieving pCR is essential for breast cancer patients to adjust the follow-up adjuvant treatment plan (65). After neoadjuvant treatment, the tumor has many changes in gross and histology, which brings specific difficulties to the pathological evaluation of post-operative specimens. Therefore, detailed, standardized, and complete pathological evaluation results can help clinicians more accurately determine the patient’s condition, formulate diagnosis and treatment plans, and provide a basis for clinical follow-up treatment and patient prognosis evaluation.

### 3.1 Histopathological evaluation

At present, the most widely used clinical pathology evaluation system is the Miller-Payne system, which is based on the number of tumor cells in the specimen before tumor treatment, determines the number of residual tumor cells in the pathological specimen unit after surgery, and calculates the tumor cell reduction ratio (**Table 2**) (66). After neoadjuvant chemotherapy, most of the cancer cells showed degenerative changes. The specific manifestation was that the morphology of the cancer cells was irregular, the whole cell was swollen, and the boundary was unclear. It could be observed that vacuoles appeared in the cytoplasm, and the nucleus was enlarged or showed deep staining and deformity (67). The cases diagnosed as pCR by postoperative histopathology have been confirmed by multiple studies (68), and their recurrence-free survival rate and overall survival rate are better than those of cases that have not achieved complete pathological remission.

**Table 2:** The interpretation of the Miller-Payne system.

MP grades	Mammograms’ results
Grade 0	No change or some alteration to individual malignant cells but no reduction in overall cellularity.
Grade 1	A minor loss of tumor cells but overall cellularity still high; up to 30% loss.
Grade 2	Between an estimated 30% and 90% reduction in tumour cells.
Grade 3	A marked disappearance of tumor cells such as only small clusters or widely dispersed individual cells remain; more than 90% loss of tumor cells.
Grade 4	No malignant cells identifiable in sections from the site of the tumor; only vascular fibroelastotic stroma remains often containing macrophages. However, ductal carcinoma *in situ* (DCIS) may be present.

### 3.2 Tumor biomarkers

Breast cancer is divided into four different molecular types (Luminal A, Luminal B, Triple Negative, and HER positive) according to its hormone receptor status, human epidermal growth factor receptor 2 (HER2), and Ki-67 expression. Breast cancer patients of different types show different responsiveness to chemotherapy drugs. Immuno-histochemistry technology uses the combination of antigens and specific antibodies to observe the distribution of antigen-antibody complexes in tissues under the microscope and can perform qualitative, localized, and quantitative analysis of the corresponding protein molecules thereby assessing the efficacy of NAC (65). The immunohistochemical results of conventional breast cancer should include such as estrogen receptor (ER), progesterone receptor (PR), human epidermal growth factor receptor (HER-2), cell proliferation marker ki-67, oncogene, and its expressed proteins (p53, BRCA1, BRCA2). At this stage, immunohistochemistry has been widely used in the pathological diagnosis of breast cancer. It can help determine the molecular classification of breast cancer and is an indispensable evaluation factor for diagnosing and treating breast cancer.

#### 3.2.1 Estrogen receptor, progesterone receptor

Among various hormones, ER is closely related to the occurrence and development of breast cancer, and ER and PR are involved in regulating the proliferation, differentiation, and growth of breast cells and tumor cells (69). At present, scholars are paying more attention to ER research because ER’s expression status is of great significance to the selection and prognosis of endocrine therapy. It is also an indicator of whether NAC is sensitive or not. Tumor cells with ER-positive are generally better in differentiation, distant metastases occur more slowly, and patient survival prognosis will be better. Patients with ER-negative expression are more susceptible to NAC than ER-positive expression. The reason may be that ER-negative tumor cells have strong proliferation ability and poorer cell differentiation, while poorly differentiated tumor cells are more sensitive to chemotherapy (70). Some studies have shown (71, 72) that the probability of ER from negative to positive after NAC is greater than the probability of positive to negative, and an important indicator of whether endocrine therapy is effective is whether ER is positive. Therefore, the expression status of ER can be used as an essential factor to predict the efficacy of NAC, but at present, whether NAC affects the status of ER and PR is still controversial, and its clinical value still needs further research.

#### 3.2.2 Human epidermal growth factor receptor-2

About 20% of breast cancer patients have HER-2 overexpression, and despite recent advances in the development of anti-HER2 drugs, these tumors still exhibit a high proliferation rate and have a poor prognosis (73). For HER-2 detection, some studies have shown that NAC does not affect the HER2 expression status of breast cancer patients after fluorescence *in situ* hybridization (FISH), although a few patients showed alterations in immunohistochemistry before and after NAC (74). Therefore, when HER-2 immunohistochemistry is positive after chemotherapy, especially in patients with alterations compared to pre-chemotherapy, FISH should be performed to avoid false-positive diagnostic results.

#### 3.2.3 Cell proliferation marker ki-67

Ki-67 reflects the cell proliferation index, which is only expressed in the nucleus of cells in the division phase, and is absent in cells in the quiescent phase of division, so its expression level is used as an essential indicator for evaluating tumor cell proliferation and invasiveness (75). The decrease in Ki-67 expression after NAC is related to the necrosis of tumor cells and the decrease in the number of tumor cells in the division phase. Obtaining drug susceptibility information and assessing the prognosis of patients is of great significance for guiding follow-up treatment plans (76). Studies have shown that the high expression of Ki-67 is closely related to the response of neoadjuvant chemotherapy. The change of Ki-67 values before and after NAC is directly proportional to the curative effect, and low Ki-67 level has become an excellent prognostic indicator (77), but the pCR rate varies among different breast cancer subtypes. Although the conclusions of various studies are different, Ki-67 is still supported by most scholars as a predictor of NAC efficacy in some subtypes of breast cancer.

Although the predictors of NAC in breast cancer remain unclear, the status of ER, PR, HER-2, and Ki-67 indicators is useful for prediction and efficacy evaluation. Additionally, these indicators assist in drug selection. Accurate pathological assessment both before and after NAC is essential for formulating chemotherapy regimens.

### 3.3 Tumor abnormal protein

Tumor Abnormal Protein (TAP), also known as aberrant glycan glycoprotein, represents a type of abnormal glycoprotein and a calcium-histone complex. It arises from mutations in proto-oncogenes and tumor suppressor genes during the initial stages of cell carcinogenesis. The occurrence and development of TAP are often accompanied by a significant increase in TAP expression (78). Studies have shown that with the increase in tumor diameter, pathological grade, and the appearance of positive lymph nodes, ER negative, and PR negative in patients, the abnormal rate of TAP and the area of aggregates show an up-ward trend, suggesting that TAP plays a vital role in the progression of breast cancer (79). For those with better clinical efficacy, the expression level of TAP before NAC is low and the expression level of TAP is further decreased after chemotherapy, and the increase of TAP expression level is often accompanied by disease progression (80). TAP detection is a convenient operation with minimal blood sampling, reliable results, and several other advantages. Its diagnostic and preventive significance holds particular value in evaluating the efficacy of breast cancer NAC.

### 3.4 Tumor immunology

#### 3.4.1 Tumor infiltrating lymphocytes

Tumor infiltrating lymphocytes (TILs) refer to the heterogeneous lymphocyte population dominated by lymphocytes in tumor cancer nests and stroma. They are the direct response cells of the body’s immune system to the tumor’s local immune response, and they are also an essential part of the tumor microenvironment (81). Some clinical studies have shown that TILs are related to the efficacy and prognosis of neoadjuvant chemotherapy and have different roles in different subtypes. In the more aggressive subtypes of breast cancer (TNBC or Her-2(+) breast cancer), TILs have a more apparent predictive effect on the efficacy of neoadjuvant chemotherapy (82). From the current data, TILs are a quantifiable indicator. Different types of TILs have different prognostic values in breast cancer. However, it should be noted that due to the different subtypes of tumor infiltrating lymphocytes in breast cancer, the predictive function of chemotherapy efficacy is different. At present, the research on TILs is still in the stage of quantitative analysis, and the analysis of their functions still needs to be further explored. TILs may become a new factor predicting the efficacy and prognosis of breast cancer NAC in the future, providing new ideas for clinicians.

#### 3.4.2 Circulating immune cells

With the research on tumor immunity in recent years, the levels of TILs, including cytotoxic T lymphocytes and natural killer (NK) cells, are essential indicators for predicting chemotherapy sensitivity and survival (83). Breast cancer is a systemic disease. Studies have shown that there may also be a potential relationship between circulating immune cells and NAC prognosis. T lymphocytes in the blood play a significant role in immune regulation, mainly CD4+ helper T cells and CD8+ cytotoxic T cells. Clinical studies have shown that reducing or inversion of the CD4+/CD8+ ratio indicates the body’s cellular immune function disorder. Therefore, the CD4+/CD8+ ratio can be used to evaluate the body’s cellular immune function (84). In addition, some researchers have found that increasing the level of peripheral blood NK cells and maintaining the functional response of T cells to specific antigens may be related to the excellent efficacy of chemotherapy in breast cancer patients (85). Studies have shown that the high proportion of pre-NAC T cells and NK cells is an essential predictor of pCR, which may be related to the anti-tumor immunity induced by chemotherapy. In addition, the increase in the proportion of NK cells after chemotherapy also indicates a greater chance of obtaining pCR. These phenomena suggest that the status of peripheral blood lymphocytes may also be closely related to the efficacy of NAC (86). By monitoring the number and ratio change of peripheral blood lymphocyte subsets before and after NAC in breast cancer patients by flow cytometry, it can understand the immune function of breast cancer patients and make up for the lack of human factors on the evaluation of NAC efficacy. However, the exact relationship between the status of the pre-NAC lymphocyte subset and the efficacy of NAC is not fully understood. This idea still needs to be confirmed by further prospective studies.

#### 3.4.3 Circulating tumor cells

Circulating tumor cells (CTCs) are a kind of tumor cells that fall off from the primary tumor or metastasis due to diagnosis and treatment operations or spontaneous reasons and enter the peripheral blood circulation through the blood vessel or lymphatic system. Studies have confirmed that monitoring CTCs during adjuvant treatment can reflect the efficacy in time, provide a basis for evaluating the sensitivity and prognosis of the treatment plan, and help to adjust the treatment strategy in time according to the patient’s situation, and select the best treatment plan, timing and intensity (87, 88). However, whether the change of CTCs after breast cancer NAC is related to the efficacy of NAC is still controversial. Relevant studies have initially shown that changes in the number of CTCs have a special relationship with the efficacy of NAC. After NAC, the positive rate and value of CTCs are significantly reduced. Compared with the pathological gold standard, CTCs can reflect the efficacy of NAC to a certain extent (89). While analysis of CTCs offers a reproducible and minimally invasive approach, acting as a surrogate for tumor tissue, to dynamically monitor tumor genomic alterations and promptly identify drug resistance and novel therapeutic targets (72), its clinical utility remains circumscribed due to the scarcity of CTCs and a paucity of evidence for CTCs-guided interventions. The potential role of CTCs in assessing the therapeutic efficacy of breast cancer NAC warrants further elucidation through prospective, large-scale, multicenter clinical trials.

### 3.5 Tumor microenvironment

The internal environment of tumor cells is termed the tumor microenvironment, crucial for tumor cell growth, invasion, and metastasis (90). The tumor microenvironment plays a vital role in the onset, progression, metastasis, and recurrence of breast cancer. Central to the tumor microenvironment are the host’s immune and systemic inflammatory responses. The latter can amplify the aggressiveness of tumor cells and diminish their treatment sensitivity (91). Several inflammatory cytokines are instrumental in tumor growth, angiogenesis, and immune regulation. Research indicates that the neutrophil-to-lymphocyte ratio (NLR) and the platelet-to-lymphocyte ratio (PLR) before treatment reliably mirror the body’s inflammatory status. In non-luminal breast cancer patients, peripheral blood NLR and PLR can serve as objective markers, predicting the risk of clinical progression post-NAC (92). These patients face heightened coagulation risks, given the close ties between the coagulation system and tumor onset, progression, and metastasis. Fibrinogen, a key coagulation factor, is involved in tumor cell proliferation, migration, and angiogenesis.

Research indicates that breast cancer is a vascular-rich malignant tumor. Elevated fibrinogen levels are strongly associated with the invasion, metastasis, and prognosis of breast cancer. Patients who present with high fibrinogen levels prior to treatment often exhibit a less favorable prognosis, with an increased risk of mortality (93). Moreover, existing studies have confirmed that miRNAs are stably present in the blood circulation, exhibiting non-invasive, reproducible, and dynamic monitorability. They are regarded as stable blood biomarkers, essential for evaluating the tumor microenvironment (94). Adequate blood supply is essential for tumor cells to form lesions and metastases. Monitoring angiogenesis, blood flow obstruction, and alterations in the composition of nutrients and metabolic wastes within the blood can shed light on the survival status of tumor cells. It is imperative to note that while numerous predictors exist within the tumor microenvironment for NAC, the definitive clinical implications of these predictors necessitate further prospective studies.

## 4 The progress of new technology

In the era of precision medicine, early evaluation of NAC efficacy for breast cancer has emerged as a prominent trend. While a multitude of methods exist, traditional diagnostic techniques, such as color Doppler ultrasound, remain foundational. Each approach has its merits and limitations. As new technologies for assessing NAC efficacy continue to emerge, their clinical value awaits validation.

### 4.1 New ultrasound imaging system

Tumor angiogenesis plays a vital role in the occurrence and development of breast cancer, and changes in its physiological characteristics often precede anatomical changes. The blood vessels in breast cancer exhibit a networked structure. Owing to the robust metabolism and high oxygen consumption of cancer cells, tumors characteristically display a phenomenon marked by high blood content coupled with reduced oxygen levels. The concentration of hemoglobin and deoxyhemoglobin in the tumor mirrors the vascular density within and surrounding the tumor (95). The optical tomography image ultrasonography system (OPTIMUS) integrates both an ultrasound imaging subsystem and a light scattering imaging subsystem. This system captures the image details and metabolic status of breast masses, further allowing the generation of a comprehensive diagnostic index (synthesis diagnostic index, SDI) for breast lesion assessment. In principle, the system can gauge parameters like total hemoglobin and deoxyhemoglobin in tumor tissue, indirectly reflecting the activity of tumor blood vessels. A salient feature of OPTIMUS is its capability to non-invasively measure and monitor local blood parameters (96), facilitating early and molecular-level assessments of NAC efficacy. However, OPTIMUS does present certain limitations. For instance, studies have indicated discrepancies in diagnosing superficial or small tumors, leading to elevated SDI values. Furthermore, any light leakage during data acquisition can lead to erroneous results (97). The combination of other imaging tests can improve the accuracy. In real-world clinical practice, while OPTIMUS holds promise for early evaluation of breast cancer NAC efficacy, it remains prudent to withhold definitive judgments due to limited clinical validation; its limitations are yet to be addressed.

Similarly, diffuse spectral imaging technology represents another near-infrared optical imaging method grounded in visualizing tissue hemodynamics, providing insights into the state of tissue microvessels. Research has established that this technology is capable of quantifying changes in water and lipid content, which have been shown to correlate with chemotherapy efficacy (98). Quantitative ultrasound is a tissue characterization technology that can detect the ultrasound radio frequency signal of the tissue reverse radiofrequency. It can reflect the effectiveness of NAC by monitoring the apoptosis of early tumor cells in the treatment. Quantitative ultrasound and diffuse spectral imaging parameters are statistically significant for judging the pathological response after one cycle of NAC (99). Quantitative ultrasound and diffuse spectral imaging are non-invasive examinations, cost-effective, and provide information about metabolism, physiological characteristics, and biological activity. However, their application in predicting and monitoring the NAC curative effect still needs further research.

### 4.2 Single-cell sequencing

Triple-negative breast cancer (TNBC) is characterized by the absence of estrogen receptor (ER), progesterone receptor (PR), and human epidermal growth factor receptor 2 (HER-2) expression. Studies have shown that TNBC patients have high somatic mutations, frequent TP53 mutations, and complex aneuploidy rearrangements, leading to extensive intratumoral heterogeneity (100). Compared to other breast cancer subtypes, TNBC patients typically present with larger primary tumors at diagnosis and exhibit a higher degree of malignancy. Lacking specific therapeutic targets, TNBC does not respond well to hormone or targeted therapies. Presently, treatment options predominantly rely on chemotherapy-based systemic approaches. Nevertheless, approximately 50% of TNBC patients demonstrate resistance to NAC, contributing to the refractory nature of the disease (101).

In recent years, with the emergence of high-throughput sequencing technologies, single-cell sequencing (SCS) has evolved and become an optimal method for probing the intricacies of TNBC tumors. SCS allows for the acquisition of genomic, transcriptomic, and epigenetic data from individual cells, effectively highlighting the unique mutational phenotypes found in single tumor cells. This technology serves as a powerful tool in addressing intratumoral heterogeneity, reconstructing evolutionary lineage, and identifying rare cell subpopulations. It offers promising avenues for refining the precision treatment strategies for TNBC (102, 103).

Studies have shown that the application prospects of SCS in TNBC’s NAC include: (1) determination of tumor subtypes, grouping, and precise treatment; (2) revealing the mechanism of resistance and monitoring drug response; (3) clarifying the mechanism of metastasis and discovering therapeutic targets; (4) exploring immunity evolution, evaluation of treatment effect.

However, SCS technology and associated methodologies present certain challenges:1) In the process of single-cell separation and extraction, it is still a challenge to accurately screen target cells and prevent contamination; 2) In the process of target molecule amplification and sequencing, uneven coverage, the presence of noise, and inaccurate quantification of sequencing data occur from time to time (104). While SCS technology offers promising advancements for monitoring NAC efficacy in TNBC patients, its widespread clinical adoption is hindered by the substantial costs associated with bioinformatics analysis. Such financial burdens can be particularly challenging for patients already grappling with the weight of their disease. As the medical community increasingly prioritizes individualized treatment, the role of NAC in TNBC becomes even more crucial. Employing SCS technology in this domain holds the potential to usher the personalized treatment of TNBC into a new era.

### 4.3 Serological indicators

With the in-depth research on breast diseases, NAC has become the first choice treatment for patients with locally advanced breast cancer, inflammatory breast cancer, and breast-conserving patients. At present, the efficacy evaluation of NAC mainly focuses on clinical imaging examination and invasive histopathological evaluation (105). In contrast, serological indicators have the advantages of convenient material extraction, slight body trauma, and repeatable monitoring. Therefore, effective serological indicators are of great benefit in assessing the efficacy of neoadjuvant chemotherapy. Peripheral blood vascular endothelial growth factor (VEGF) is a specific mitogen for vascular endothelial cells derived from arteriovenous and lymphatic vessels. Studies have shown that serum VEGF is an effective indicator for the early diagnosis of breast cancer (106). In clinical practice, high expression of serum VEGF is related to poor prognosis, but there are few reports about the use of serum VEGF to evaluate the efficacy of neoadjuvant chemotherapy. CTCs are a kind of tumor cells that fall off from the primary tumor or metastasis due to diagnosis and treatment operations or some spontaneous reasons and enter the peripheral blood circulation of the body through the blood vessel or lymphatic system. Previous studies have confirmed that CTCs can be used as an independent indicator of disease progression and overall survival prognosis in patients with metastatic breast cancer (107). Studies have confirmed a correlation between serum VEGF levels and CTCs levels (108). As the most potent vascular growth-stimulating factor known so far, VEGF directly participates in tumor angiogenesis, promoting tumor growth and metastasis. CTCs are a real-time “liquid biopsy” marker (109). Combining the “Tumor Vascular Regulation Theory” and CTCs, the formation of CTCs is closely related to the blood vessel and lymphatic system. VEGF directly participates in the formation of blood vessels, enhances vascular permeability, and accelerates the formation of lymphatic vessels (110). Highly expressed VEGF provides the vascular and lymphatic systems needed for CTCs to shed. In summary, changes in serum VEGF levels and CTCs levels can be used to evaluate the efficacy of neoadjuvant chemotherapy, adding a new option for the accurate evaluation of neoadjuvant efficacy.

### 4.4 Metabolite

Current studies have found that some breast cancer patients have low sensitivity to NAC or even no effect, and delays in other treatments due to chemotherapy and adverse (111) effects may even increase the risk of death for patients. Therefore, it is urgent to find a factor that can predict the sensitivity of chemotherapy to reduce the failure rate of treatment. There are many methods to evaluate the effect of chemotherapy in the clinic, but there are certain shortcomings in imaging examination and pathological examination. Soluble E-cadherin (sE-cadherin) is a soluble fragment produced by the cleavage of E-cadherin. Following carcinogenesis, the amount of sE-cadherin in the patient’s serum and urine increases considerably, influencing early-stage tumor identification and critical in clinical illness assessment (111). sE-cadherin is an extracellular fragment formed by the degradation of E-cadherin by a variety of proteases. The amount of sE-cadherin in healthy people’s serum and urine is extremely low, whereas the amount of sE-cadherin in stomach cancer, prostate cancer, and other malignancies is high (112). Existing studies have explored the relationship between sE-cadherin and various malignant tumors and believe it can become a new tumor marker for diagnosis, efficacy, and prognostic evaluation (113).

Clinical studies have shown that the expression level of sE-cadherin in the peripheral blood of cancer patients is significantly increased. In contrast, the level of sE-cadherin in the serum of healthy people is very low. Moreover, sE-cadherin is associated with pathological features such as TNM staging, tumor size, and pathological tissue grading in breast cancer patients (114). As a metabolite, sE-cadherin is easy to collect, no biopsy is required, and the detection method is simple and mature. It suggests that metabolites can be used as an indicator or can be used as a diagnostic indicator for breast cancer screening, providing important reference information for the prognosis evaluation of breast cancer patients. However, there are few studies on metabolomics in breast diseases, and it is expected to become a new research hotspot in the future.

## 5 Conclusion and outlook

Breast cancer treatment has progressed from primary surgical treatment to a complete whole-body therapy involving surgery, chemotherapy, and targeted therapy, marking a significant milestone in medical history. Unfortunately, so far, a large number of clinical trials have confirmed that for most patients, neoadjuvant chemotherapy cannot effectively prolong the disease-free survival rate and overall survival rate of breast cancer patients. However, NAC is reducing the tumor volume to increase the breast-conserving rate. It is of great significance to downgrade locally advanced inoperable patients to provide surgical opportunities and evaluate the sensitivity of tumor cells to chemotherapy drugs. Neoadjuvant therapy has different treatment plans and cycles for breast cancer patients of different molecular types. During the treatment process, close monitoring and timely assessment of the efficacy of chemotherapy are vital. Standardized efficacy evaluation can grasp the current tumor burden of breast cancer patients, guide the next step of the treatment plan, and find an accurate time window before each step of the treatment decision.

At present, the evaluation of the efficacy of neoadjuvant therapy for breast cancer includes two aspects: non-invasive clinical evaluation and invasive tissue pathological evaluation. Clinical evaluation is the evaluation system that indirectly measures the size changes of solid tumors through imaging or physical examination, while histopathological evaluation system is the evaluation of obtaining pathological specimens of the lesion tissue through methods such as surgery or needle biopsy and observing the evaluation of tumor cell residues. Imaging examinations include breast X-ray examination, breast ultrasound examination, and breast MRI examination, but none of them can distinguish between necrotic tissue and fibrous scar tissue after chemotherapy, and the accuracy rate is low. Clinical evaluation is affected by subjective factors such as the experience of clinical physicians, and the evaluation is prone to deviations, and there are drawbacks that the curative effect cannot be accurately evaluated. Histopathological evaluation is the gold standard for evaluating tumor response after chemotherapy. The diagnostic accuracy is reliable, but it needs to be performed after NAC and surgery. It has a noticeable lag and cannot understand the sensitivity of chemotherapy drugs in time, and it is not easy to adjust the chemotherapy regimen in time. It is easy to miss the best time to adjust the plan. Therefore, it is crucial to find new indicators that can respond to the curative effect on time and be tested repeatedly to monitor it.

In summary, NAC is an essential treatment for breast cancer. Early evaluation of its efficacy has become a hot trend in today’s precision medicine era. Although there are many methods, various methods have their pros and cons. The evaluation of new technologies still needs a large number of clinical verifications. An accurate assessment of the stage of disease development is conducive to the development of individualized treatment plans and effectively curbs the overtreatment phenomenon that prevails at this stage. In order to seek more convenient, more sensitive, and more specific disease diagnosis and evaluation methods, the author believes that the improvement and improvement of NAC efficacy evaluation can be considered from the following aspects, physical evaluation of blood vessel growth, tumor size, and other indicators, functional evaluation of tumor growth, metabolism and other indicators, and supplementation of imaging examination to improve the accuracy of lesion judgment. NAC can be better used in clinical treatment by studying tumor molecular biology in large sample cases. Therefore, while developing new technologies, in the process of clinical application, macroscopic imaging must be combined with microscopic biomarkers, and substantive indicators must be combined with functional indicators to establish a more scientific and efficient system of disease treatment evaluation.

## Author contributions

There was equal contribution from YC and YQ to the manuscript. The manuscript was written by YQ and YC after reviewing the literature. YC and YQ drafted and revised table. YC designed and made the figure. Manuscript design and revision were done by KW. All authors contributed to the article and approved the submitted version.
